# Morphological and Molecular Characterization of a Novel Fungal-feeding Stem Nematode *Ditylenchoides agaricivorus* n. sp. (Tylenchida: Anguinidae) from Intercepted Samples

**DOI:** 10.2478/jofnem-2025-0003

**Published:** 2025-02-27

**Authors:** Che-Chang Liang, Pei-Chen Chen

**Affiliations:** Department of Plant Pathology, College of Agriculture and Natural Resources, National Chung Hsing University, No. 145, Xingda Rd., South Dist., Taichung City 402, Taiwan (R.O.C.)

**Keywords:** *Ditylenchoides*, fungal-feeding, ITS, molecular phylogeny, morphology, morphometrics, new species, partial LSU, taxonomy

## Abstract

A new species of the genus *Ditylenchoides*, *D. agaricivorus* n. sp., collected from coconut fiber used as growing media for staghorn ferns and intercepted during import quarantine, is described and illustrated herein based on morphological and molecular studies. The new species is characterized by a body length of 728 (612–846) μm and 641 (511–720) μm in female and male, respectively, delicate stylet 8.0 (7.4–8.4) μm long, six lines in the lateral field, median bulb of esophagus well-developed, muscular with crescentic valve, post-vulval uterine sac well-developed, 36 (22–52) μm long, female tail elongate-conoid with finely rounded terminus. The results of phylogenetic analyses based on sequences of the D2D3 expansion region of 28S rRNA and ITS confirmed the close molecular relationship between *D. agaricivorus* n. sp., and other *Ditylenchoides* species such as *D. africanus*, *D. arachis*, *D. destructor*, *D. halictus*, *D. myceliophagus* and *D. persicus*. *Ditylenchoides agaricivorus* n. sp. was successfully reared on the *Rhizoctonia solani* and *Agaricus biporus*. However, *D. agaricvorus* n. sp. did not reproduce when culturing on *Lentinula edodes*, *Pleurotus erungii*, *Volcariella volvacea*, *A. bitorquis*, nor on callused carrot disks, and alfalfa seedlings.

*Ditylenchus* Filipjev, 1936, is a taxonomically complex genus of plant-parasitic nematodes and one of the most challenging genera in nematode taxonomy. Currently, *Ditylenchus* is classified within the family Anguinidae or the superfamily Anguinoidea (Siddiqi, 2000). Over the years, various concepts have been proposed to define this genus and elucidate its evolutionary trends (Paramonov, 1970; Brzeski, 1981, 1991; Fortuner, 1982; Subbotin and Ryss, 2024). Paramonov (1970) proposed dividing the genus into two groups: pathogen-specific species and non-specific pathogenic or fungal-feeding species. Skarbilovich (1980) classified the genus into the “dipsacoid” group (*D. dipsaci*) and the “destructoid” group (*D. destructor*). Fortuner and Maggenti (1987) broadly defined *Ditylenchus*, recognizing 91 valid species and considering *Nothotylenchus* Thorne, 1941, *Boleodoroides* Mathus, Khan and Prasad, 1966, *Diptenchus* Khan, Chawla and Seshadri, 1969, *Safianema* Siddipi, 1980, and *Orrina* Brzeski, 1981 as synonyms. They further categorized the genus into three groups: species parasitizing above-ground plant parts, species with degenerated esophageal median bulbs, and species associated with bark beetles. Siddiqi (2000) defined the genus more precisely, classifying 57 species into two groups based on morphology and feeding preferences. The *D. dipsaci* group is characterized by four lateral field incisures, sharply pointed tail tips, and a preference for plant cell feeding, with diminished fungal-feeding traits. The *D. triformis* group includes primarily fungal-feeding species with six lateral field incisures and rounded tail tips (Paramonov, 1970; Skarbilovich, 1980; Fortuner and Maggenti, 1987; Siddiqi, 2000).

Subbotin and Ryss (2024) redefined the genus *Ditylenchus*, proposing a comprehensive definition and establishing two new genera: *Ditylenchoides* Subbotin and Ryss, 2024 and *Paraditylenchus* Subbotin and Ryss, 2024. *Ditylenchoides* genus, with *Ditylenchoides destructor* Thorne, 1945 as the type species, includes 14 additional species. *Paraditylenchus* genus, with *Paraditylenchus gallaeformans* (Oliveira et al., 2013) as the type species, was also established. *Ditylenchoides* differs from *Ditylenchus* in body length, the number of lateral field incisures, tail terminus shape, and the types of plant symptoms induced. *Paraditylenchus* differs from *Ditylenchus* in body length, post-vulval uterine sac length, and bursa length. Additionally, *Ditylenchus drepanocercus* was reassigned to *Zeatylenchus* (Zhao et al., 2013) as *Zeatylenchus drepanocercus* (Goodey, 1958; Subbotin and Ryss, 2024).

The previous Ditylenchus spp. are conserved in gross morphology, making species identification very difficult (Brzeski, 1991). In recent years, phylogenetic inference has proven to be an effective method to differentiate species of the morphologically conservative previous genus *Ditylenchus* (Subbotin et al., 2005; Vovlas et al., 2011, 2016).

A population of *Ditylenchoides* was collected by the Animal and Plant Health Inspection Agency, Council of Agriculture, Executive Yuan, Taiwan, from coconut fiber used for growing staghorn ferns, intercepted during import quarantine. Morphological and morphometric studies revealed clear differences between this population and other known *Ditylenchoides* species. Several studies have reported that species of *Ditylenchoides* such as *Ditylenchoides arachis* Zhang, Liu, Janssen, Zhang, Xiao, Li, Couvreur and Bert 2014, *D. destructor, Ditylenchoides halictus* Giblin-Davis, Erteld, Kanzaki, Ye, Zeng and Center, 2010, and *Ditylenchoides myceliophagus* Goody, 1985 feed and reproduce on fungal hyphae under artificial cultural conditions (Goodey, 1960; Hussey and Krusberg, 1971; Latif and Mian, 1995; Giblin-Davis et al., 2010; Zhang et al., 2014).

Thailand is abundant in tropical plant resources, with its germplasm exchange with Taiwan primarily focusing on fruit trees, orchids, ornamental plants, and ferns (Kuo and Chiu, 2013). Elkhorn fern, known for its elegant and variable forms, is a popular plant for households in Taiwan (Chang et al., 2012). During quarantine procedures, an unknown nematode species was isolated from the growing medium (coconut fiber) for elkhorn ferns. The objectives of this research were: *i)* to describe the taxonomic status of new species *D. agaricivorus* n. sp.; *ii)* to determine the molecular phylogenetic affinities of *D. agaricivorus* n. sp. with closely related species using D2D3 of 28S and ITS rRNA gene sequences; and iii) to determine the host status of several economically important mushrooms of the *D. agaricivorus* n. sp, and to evaluate whether it could feed on plants.

## Materials and Methods

### Nematode populations

Nematodes were extracted from the imported coconut fiber used for growing staghorn ferns, intercepted by the Animal and Plant Health Inspection Agency (APHIA), Council of Agriculture, Executive Yuan, Taiwan, on July 25, 2019, using the modified Baermann funnel method for 24 h (Wu et al., 2010). Because the preliminary morphological identification was consistent with the morphological characteristics of the genus *Ditylenchoides*, such as having six lateral lines, the nematodes in this sample were transferred into the fungal culture. Meanwhile, one female and one male were picked from that sample and transferred onto *Rhizoctonia solani* Kühn PDA slant to establish a pure population for morphological identification and molecular characterization.

### Morphological identification

Nematode samples were prepared for microscopic observation, according to De Grisse (1969). Nematodes were handpicked under a dissecting microscope (SZX16, Olympus, Japan), killed by adding hot 4% formalin solution, transferred to anhydrous glycerol, and mounted on permanent slides. Morphology and morphometrics of holotype and paratype nematodes were observed using a light microscope (BX53, Olympus, Japan). Photographs of nematodes were taken by a digital camera attached to the same microscope, and drawings were made using Adobe Photoshop Sketch (Adobe, San Jose, USA). Permanent slides were deposited in the USDA Nematode Collection, Beltsville, MD, USA, and the Plant Parasitic Nematodes Laboratory, National Chung Hsing University, Taichung, Taiwan.

### Molecular identification

Four nematodes were transferred into a 0.2 ml PCR tube containing 8 μl of DirectPCR^®^ Lysis Reagent Tail (Viagen Biotech, Los Angeles, USA). After adding 1 μl proteinase K and dithiothreitol, samples were incubated at 65°C for 20 min, heated at 95°C for 10 min, and stored at −20°C until use. Two microliters of DNA were transferred to a PCR tube containing 2 μl of 10X Gen TaqPlus buffer (100 mM Tris-HCl, pH 9.0, 500 mM KCl, 0.1% gelatin, 20 mM MgCl2, 1% Triton X-100), 2 μl of 2.5 mM dNTPs, 1 μl of each primer (10 mM), 0.2 μl of GenTaq plus DNA polymerase (GeneMark, Taichung, Taiwan) and distilled H_2_O to make up a final volume of 20 μl. The ITS region was amplified using forward primer TW81 (5′-GTTTCCGTAGGTGAACCTGC-3′) and reverse primer AB28 (5′-ATATGCTTAAGTTCAGCGGGT-3′) as described by Subbotin et al. (2005). The D2D3 expansion segment of 28S rRNA was amplified using primers D2A (5′-ACAAGTACCGTGAGGGAAAGTTG-3′) and D3B (5′-TCGGAAGGAACCAGCTACTA-3′) (De Ley et al., 1999).

PCR conditions for both ITS and D2D3 regions were as follows. Reactions started with an initial denaturation at 94°C for 7 min, followed by 35 cycles of denaturation at 94°C for 50 s, annealing at 55°C for 50 s, and extension at 72°C for 1 min, and a final step at 72°C for 7 min. PCR products were subjected to electrophoresis in a 1.2% agarose gel, and the product on the gel was collected using a DNA clean/extraction kit (Gene Mark, Taichung, Taiwan). Purified ITS and D2D3 fragments were independently cloned to a pCR^®^ 2.1-Topo^®^ vector using TOPO TA cloning^®^ kit (Invitrogen, Waltham, USA) and sequenced by the Automated DNA sequencing Service Laboratory (BTC of NCHU, Taiwan). Sequences of the newly identified *Ditylenchoides* sp. were obtained from three generations of nematode populations. For each sequencing, three clones were sequenced, resulting in nine sequences. These sequences were aligned and confirmed, forming a consensus sequence before being deposited in the National Center for Biotechnology Information (NCBI) database under the accession numbers MW042406 for the ITS rRNA gene and MW042403 for the 28S D2D3 gene.

### Phylogenetic analysis

Sequences of 28S D2D3 and ITS of *Ditylenchoides* sp. were aligned using MEGA 7 (Kumar et al., 2016) with default parameters, including sequences of other Ditylenchus, Ditylenchoides, Paraditylenchus, and *Zeatylenchus* nematodes available in GenBank to construct phylogenetic trees. Sequences of *Zeldia punctata* (Thorne, 1925, 1937) and *Acrobeloides camberenensis* (Khan, 1992; Siddiqi, 2000; De Ley, 2005) were used as outgroups of 28S D2D3 analysis according to Subbotin and Ryss, (2024), while *Bursaphelenchus xylophilus* (Steiner and Buhrer, 1934; Nickle, 1970) was used as the outgroup of ITS analysis according to Vovlas et al., (2016). Phylogenetic analyses were performed based on Bayesian inference (BI) using MrBayes 3.1.2 (Ronquist and Huelsenbeck, 2003). D2D3 and ITS sequences were processed for BI analysis under the GTR + I + G model and ran with 1 × 10^6^ generations setting the burn-in at 1000. Markov Chain Monte Carlo (MCMC) methods were used within a Bayesian framework to estimate the phylogenetic tree’s posterior probabilities (pp). Trees were visualized using Fig Tree software (v1.4.3, Molecular Evolution, Phylogenetics, and Epidemiology).

### Host test

*Rhizoctonia solani* was used as a positive control. Four economically important mushrooms *Lentinula edodes* (Berkeley) Pegler, *Pleurotus erungii* (de Candolle) Gillet, *Volcariella volvacea* (Bulliard) Singer, *Agaricus biporus* (Lange) Imbach and *Agaricus bitorquis* (Quélet) Saccardo were used as food sources to examine their ability to support the reproduction of *Ditylenchoides* sp. *Rhizoctonia solani* was originally isolated from okra, *Abelmoschus esculentus* (Linnaeus) Mönch, and stored in the Plant Parasitic Nematodes Laboratory of the Department of Plant Pathology at NCHU. Mushroom isolates were obtained from the Edible and Medicinal Mushroom Laboratory at Taiwan Agricultural Research Institute, Ministry of Agriculture. An agar plug containing fungal mycelium was transferred to a 4.5-cm Petri dish filled with straw compost medium (25 g dry straw compost, 50 g maize crumble, 17 g agarose per liter), and the dish was incubated at 25°C for 14 days. When the plate was filled with mycelium, 15 females and 5 males of *Ditylenchoides* sp. were handpicked and transferred to the fungal plate, and the lids were sealed with parafilm (Bemis, Neenah, USA). Fungal cultures without *Ditylenchoides* sp. were used as a negative control. The plates were cultured at 25°C in the dark for 21 days. The experiments were performed three times, and all the treatments had 4 replicates.

Besides mushrooms, callused carrot disks and alfalfa seedlings were included in the host tests. With some modifications, carrot disks were prepared as described by Kaplan and Davis (1990). Fresh carrots were purchased from a local grocery store, thoroughly washed with tap water, and surface-sterilized with 0.05% NaOCl solution (The Clorox Company, Oakland, USA) for 30 min, then sprayed with 75% ethanol. Carrots were sliced along vascular cambium to produce 5 mm diameter in length and 5 mm thick disks. Two disks were transferred onto plates containing 1.7% Bamborg B-5 medium (PhytoTechnology Laboratories, Lenexa, USA) and incubated at 25°C in the dark for 30 days until the formation of callus on the surface of carrot disks. Two hundred surface-sterilized all-stages of *Ditylenchoides* sp. in 100 μl sterile water were transferred to the margins of carrot callus, and lids were sealed with parafilm to maintain moisture. The disks were incubated at 25°C in the dark for 35 days and the reproduction factor (Rf) values were recorded.

Seeds of alfalfa were surface-sterilized in a 0.05% NaOCl solution for 30 s, transferred to 75% ethanol for 30 s, and rinsed with sterile distilled water. Seeds were placed on petri dishes containing 1.7% Bamborg B-5 medium and incubated at room temperature. After alfalfa cotyledon was fully expanded (~3–5 days), a sterile cotton ball was placed on the cotyledon, and two hundred surface-sterilized all-stages of *Ditylenchoides* sp. in 100 μl sterile water were transferred to the cotton ball. The plate was sealed with parafilm to maintain moisture. At the end of each experiment, the medium of fungal cultures was cut into small pieces, and the vermiform life stages of the nematodes were recovered using the modified Baermann funnel method for 48 hours. The numbers of all stages of *Ditylenchoides* sp. were counted with the aid of a dissecting microscope. Callused carrot disk and alfalfa cotyledon were stained following the method of Bybd et al. (1983). Callused carrot disk and entire alfalfa plants were soaked in 30 ml of bleach solution (5.25% NaOCl) for 15 min, rinsed with tap water, transferred to 30 ml of water, and incubated for 15 min. Plant tissues were placed in 30 ml cotton blue solution [1 ml stock solution (0.35 g of cotton blue powder dissolved in 250 ml of lactic acid and 750 ml of distilled water) in 29 ml water] and microwaved for 30 s or until the solution started boiling. Plant tissues were de-stained by rinsing them in tap water to remove excess stain and soaking them in nonacidified glycerin. The plant tissues and glycerin were microwaved for 30 s or until glycerin began boiling. After the glycerin cooled to room temperature, plant tissues were observed with a light microscope. The number of nematodes was counted and the reproductive factor (Rf) was calculated in all potential host experiments. The experiments were performed three times and both treatments with 3 replicates.

## Results

### Systematics

**Figure 1:**
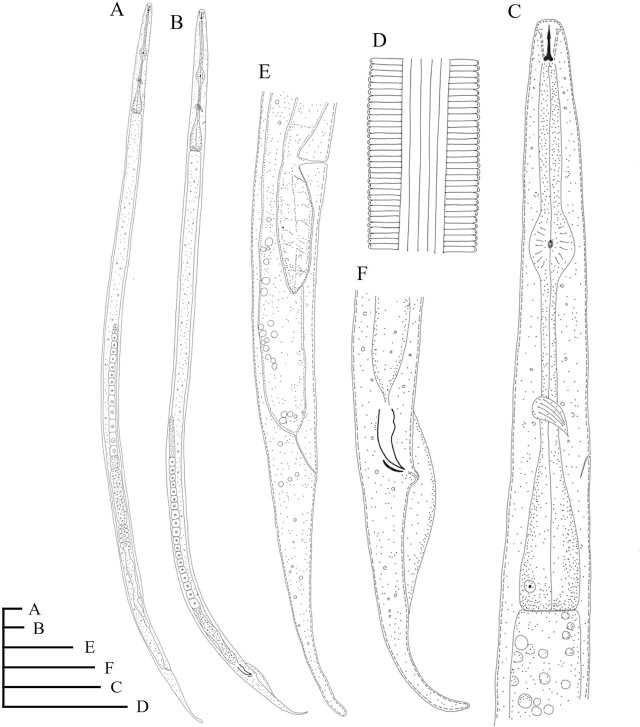
The line drawing of *Ditylenchoides agaricivorus* n. sp. A: Female entire body; B: Male entire body; C: Female esophageal region; D: Lateral field at mid-body; E: Female posterior body region; F: Male posterior region. (Scale bars = 20 μm).

**Figure 2:**
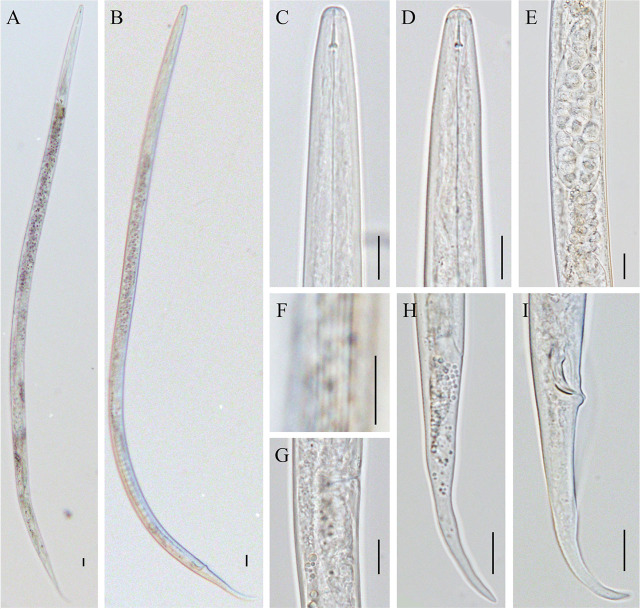
Light micrographs of *Ditylenchoides agaricivorus* n. sp. A: Female entire body; B: Male entire body; C, D: Female anterior body region; E: Spermatheca; F Lateral field at mid-body; G: Vulval region and post-vulval uterine sac; H: Female tail region; I: Male tail region. (Scale bars = 20 μm).

*Ditylenchoides agaricivorus n. sp.*

(Table 1; Figs. 1 and 2)

**Table 1:** Morphometrics of *Ditylenchoides agaricivorus* n. sp.

**Character/ration**	**Female**		**Male**
	
	**Holotype**	**Paratypes**	**Paratypes**
n	-	20	15
L	745.58 ^a^	728 ± 61.3 (612–846)	641 ± 61.7 (511–720)
a	40.85	40.4 ± 2.3 (34.5–43.4)	41.3 ± 3.1 (34.5–47.1)
b	6.83	6.4 ± 0.7 (5.4–8.5)	5.6 ± 0.4 (4.7–6.2)
c	10.82	11.1 ± 0.7 (9.9–12.7)	10.6 ± 0.9 (9.1–12.0)
c’	5.28	5.5 ± 0.6 (4.5–6.8)	5.7 ± 0.6 (4.7–6.5)
V or T (%)	82.57	80.0 ± 1.8 (75.5–82.6)	37.3 ± 3.0 (34.0–43.1)
Lip region height	2.22	2.3 ± 0.1 (2.0–2.7)	1.9 ± 0.2 (1.5–2.2)
Lip region diam.	5.83	5.8 ± 0.3 (5.4–6.4)	5.1 ± 0.3 (4.5–5.6)
Stylet length	8.14	8.0 ± 0.3 (7.4–8.4)	7.6 ± 0.3 (7.1–8.2)
Conus length	2.95	2.6 ± 0.3 (2.1–3.0)	2.6 ± 0.3 (2.2–3.1)
m	36.24	32.9 ± 3.4 (26.7–38.1)	34.0 ± 3.7 (28.8–39.6)
Body diam.	18.25	18.1 ± 2.5 (14.1–24.3)	15.6 ± 1.8 (12.7–19.4)
Nerve ring from anterior	72.7	73.6 ± 5.6 (62.9–85.0)	69.8 ± 3.1 (65.5–75.7)
Excretory pore form anterior	83.67	94.3 ± 5.0 (83.7–108.2)	88.9 ± 6.0 (71.9–98.7)
Esophagus length	109.17	113.6 ± 8.3 (91.0–127.6)	114.4 ± 4.1 (107.0–119.3)
Vulva to anus distance (VA)	80.5	79.1 ± 7.5 (66.7–90.3)	-
Post vulval uterine sac	40.76	36.5 ± 7.4 (21.8–51.6)	-
PUS/VA (%)	50.63	46.0 ± 7.6 (32.7–69.4)	-
Ovary or testis length	266.18	254.6 ± 22.6 (217.7–303.6)	238.8 ± 24.1 (190.3–287.8)
Anal (cloacal) body diam.	13.05	12.0 ± 2.0 (9.6–16.8)	10.8 ± 1.2 (9.0–12.9)
Tail length	68.92	65.5 ± 5.4 (58.2–76.9)	60.8 ± 4.3 (49.9–68.0)
Spicule length (arc line)	-	-	15.4 ± 0.6 (14.7–16.7)
Gubernaculum length	-	-	5.3 ± 0.4 (4.4–6.0)

a All measurements were in μm and in the form of: mean ± standard deviation (range).

Line drawing and light micrographs of *Ditylenchoides agaricivorus* n. sp. are shown in Figures 1 and 2, respectively. The morphometric measurements of *Ditylenchoides agaricivorus* n. sp. are listed in Table 1.

### Description

*Female*. Body slightly ventrally arcuate, with a finely annulated cuticle (Figs. 1A; 2A). Annuli are circa (ca.) 1.3–1.5 μm high at mid-body, a lateral field with six incisures (Figs. 1D; 2F). The lip region is slightly flattened, continuous with body contour, ca. 2.3 (2.0–2.7) μm high and 5.8 (5.4–6.4) μm wide. The stylet is weak and short, with the conus being ca. 26.7–38.1% of the stylet length and distinct rounded basal knobs. The orifice of the dorsal gland was close to the stylet knobs. Procorpus cylindroid, metacorpus (median bulb) elongated fusiform with crescentic valves slightly anterior to the center. Isthmus is elongated and slender, with a nerve ring located around the middle and late part of the isthmus and enveloping isthmus (Figs. 1C; 2C, D). Excretory pore (EP) opening located in the posterior region of the nerve ring, ca. 94.3 (83.7–108.2) μm from the anterior end. Terminal bulb pyriform and offset from the intestine (Fig. 1C). The vulva is a transverse slit, vagina extended into the body slightly less than half body width. Ovary monodelphic-prodelphic with a single row of oocytes (Figs. 1A; 2A). The spermatheca is long with large rounded sperm (Fig. 2E). Post-vulval uterine is sac relatively long, ca. 1.4–1.8 vulval body diam. long (Figs. 1E; 2G). Vulva-anus distance ca. 79.1 (66.7–90.3) μm. Tail about 5.5 (4.5–6.8) times the anal body width, elongate-conoid, and became narrow with a finely rounded terminus (Figs. 1E; 2H).

Male general morphology was similar to that of females except for the reproductive system. Testis single and anteriorly outstretched (Figs. 1B; 2B). Spicules paired and ventrally arcuate, rounded manubrium and a pointed distal tip. Gubernaculum arcuate. Bursa extends from the base of the spicules to approximately the midpoint between the anus and the tip of the tail (Figs. 1F; 2I).

### Type phoretic and locality

The new species was obtained from the staghorn fern coconut fiber growing medium intercepted during quarantine from Thailand.

### Type material

Thirteen paratype females (slide collection numbers T-7834p and T-7835p) were deposited in the USDA Nematode Collection, Beltsville, MD, USA, and the remaining paratypes were deposited at the Plant Parasitic Nematodes Laboratory, National Chung Hsing University, Taichung, Taiwan.

### Diagnosis and relationship

*Ditylenchoides agaricivorus* n. sp. is characterized by the following features: a delicate stylet with rounded basal knobs, a well-developed and valvate median bulb of esophagus, an excretory pore located at the posterior third of the isthmus to the anterior third of basal esophageal bulb, six lines presented in the lateral field, a vulva located at 75–82% of the body length, a relatively short post-vulval uterine sac (ca. 0.8–1.0 corresponding vulval body diam. long), relatively short spicules, a simple and ditylenchoid gubernaculum, a bursa covered 65–80% of the tail length, and an elongated-conoid tail of both sexes, slightly bent to the ventral side and ending in a finely rounded terminus.

Phylogenetical closely related species, such as *Ditylenchoides africanus* (Wendt et al., 1995), *Ditylenchoides anchilisposomus* (Tarjan, 1958), *D. arachis*, *Ditylenchoides clarus* (Thorne and Malek, 1968), *D. destructor*, *Ditylenchoides gilanicus* (Yaghoubi et al., 2018), *D. halictus*, *D. myceliophagus, Ditylenchoides persicus* (Esmaeli et al., 2017), *Ditylenchoides sarvarae* (Shokoohi et al., 2018), *Ditylenchoides stenurus* (Esmaeili et al., 2017), and *Ditylenchoides tenuidens* (Gritsenko, 1971) are all displayed morphological and morphometric differences from *D. agaricivorus* n. sp.

The new species differs from several related species in various morphological and morphometric characteristics. Compared to *D. africanus*, the new species exhibit a higher ratio of female body length to body diameter at 40 (35–43) vs 24–40, a lower ratio of female body length to esophagus length at 6.4 (5.5–8.5) vs 7.11–11.8, a higher ratio of female tail length to anal body diameter at 5.5 (4.5–6.8) vs 3.1–5.1, and a shorter spicule length of 15.4 (14.7–16.2) μm compared to 17–21 μm. Similarly, the new species differs from *D. anchilisposomus* by having a higher ratio of female tail length to anal body diameter at 5.5 (4.5–6.8) vs 2.7–5.7.

When compared to *D. arachis*, the new species has a shorter male body length of 641 (511–720) μm vs 730–1022 μm, a shorter stylet length of 8.0 (7.4–8.4) μm compared to 8.6–9.6 μm, a shorter conus length of 2.6 (2.1–3.0) μm vs 3.5–4.3 μm, a shorter post-vulval uterine sac length of 36.5 (21.8–51.7) μm vs 41–65 μm, and a shorter spicule length of 15.4 (14.7–16.2) μm compared to 16–24 μm. In contrast to *D. clarus*, the new species shows a shorter stylet length of 8.0 (7.4–8.4) μm vs 8.5–10 μm and a shorter spicule length of 15.4 (14.7–16.7) μm compared to 16–24 μm. The new species is also distinguished from *D. destructor* by its shorter female body length of 728 (613–846) μm compared to 800–1400 μm, shorter male body length of 641 (511–720) μm vs 800–1300 μm, a lower percentage of male gonad to body length at 37 (34–43) compared to 73–80, and a lower ratio of female body length to tail length at 11 (10–13) compared to 15–20.

Compared to *D. gilanicus*, the new species has a shorter male stylet conus length of 2.6 (2.2–3.1) μm vs 3.0–3.5 μm and a longer gubernaculum length of 5.3 (4.4–6.0) μm vs 3.0–4.5 μm. The new species differs from *D. halictus* by having a shorter stylet length of 8.0 (7.4–8.4) μm vs 9.0–10.0 μm and a shorter spicule length of 15.4 (14.7–16.7) μm compared to 16.5–21.0 μm. When compared to *D. myceliophagus*, the new species exhibit a higher ratio of female body length to body diameter at 40 (35–43) vs 30, a lower ratio of female body length to tail length at 11 (10–13) vs 14, and a lower percentage of male gonad to body length at 37 (34–43) vs 57.8.

From Ditylenchoides pedrami Azimi and Abdolkhani, 2022, the new species is distinguished by a shorter stylet length of 8.0 (7.4–8.4) μm vs 9–11 μm, a higher ratio of female body length to body diameter at 40.4 (35–43) vs 29.3–34.2, a lower ratio of female body length to anal body diameter at 11.1 (9.9–12.7) vs 14.5–19.8, and a higher female tail length to anal body diameter ratio at 5.5 (4.5–6.8) vs 2.4–3.2. In comparison to *D. persicus*, the new species has a shorter male lip height at 1.9 (1.47–2.2) vs 3.0–3.5, a longer stylet length of 8.0 (7.4–8.4) μm vs 6.2 (5.0–7.0) μm, and a longer post-vulval uterine sac length of 36 (22–52) μm vs 16 (14–18) μm. The new species is further distinguished from Ditylenchoides rafiqi Vyshali, Somvanshi, Islam, Kundu and Khan, 2023 by a shorter conus length of 2.6 (2.1–3.0) μm vs 5.0–5.5 μm, and from *D. sarvarae* by a shorter stylet length of 8.0 (7.4–8.4) μm vs 9–15 μm, a lower ratio of female body length to anal body diameter at 11.1 (9.9–12.7) vs 13.7–18.2, and a shorter spicule length of 15.4 (14.7–16.7) μm compared to 22–26 μm.

Finally, the new species is differentiated from *D. tenuidens* by having a shorter esophagus length of 114 (91–128) μm vs 126–148 μm, a higher ratio of post-vulval uterine sac length to vulva-to-anus distance at 46 (32.7–69.4) vs 17.5–32, a shorter cloacal diameter of 10.8 (9–12.9) μm vs 13–16 μm, and a shorter male tail length of 60.8 (50–68) μm compared to 75–96 μm.

### Phylogenetic position of *Ditylenchoides agaricivorus* n. sp.

Amplification of the D2D3 expansion segment of 28S and ITS sequences from *D. agaricivorus* n. sp. specimens yielded a single fragment of 781 bp and 834 bp, respectively. The sequences were submitted to NCBI GenBank under the accession numbers MW042403 (D2D3 28S rRNA) and MW042406 (ITS rRNA), respectively. The nucleotide BLAST search in the NCBI database against using the D2D3 28S rRNA of *D. agaricivorus* n. sp. displayed 91.53% sequence similarity to *D. persicus* (KX463285). The ITS rRNA sequence of *D. agaricivorus* n. sp. showed 84.87% sequence similarity to *D. arachis* (JX040545).

**Figure 3:**
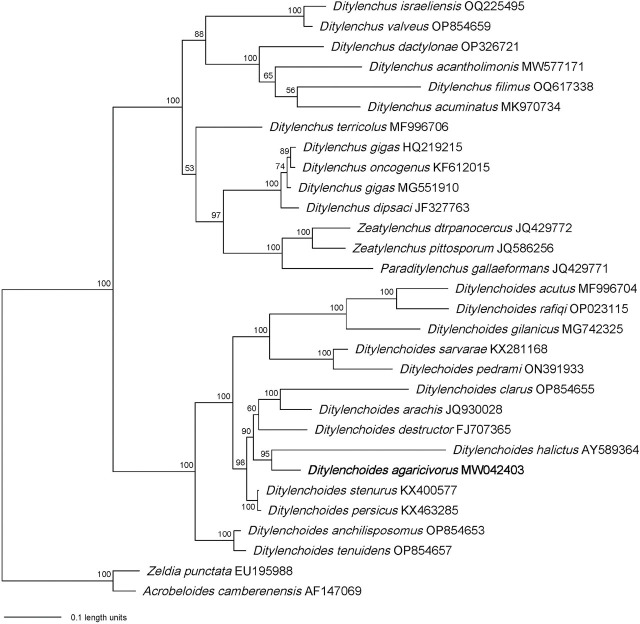
Bayesian tree generated from the D2D3 of 28S rRNA gene dataset of Anguinid nematodes with the GTR + I + G model. Newly obtained sequence in bold letters (Scale bar = expected changes per site).

**Figure 4:**
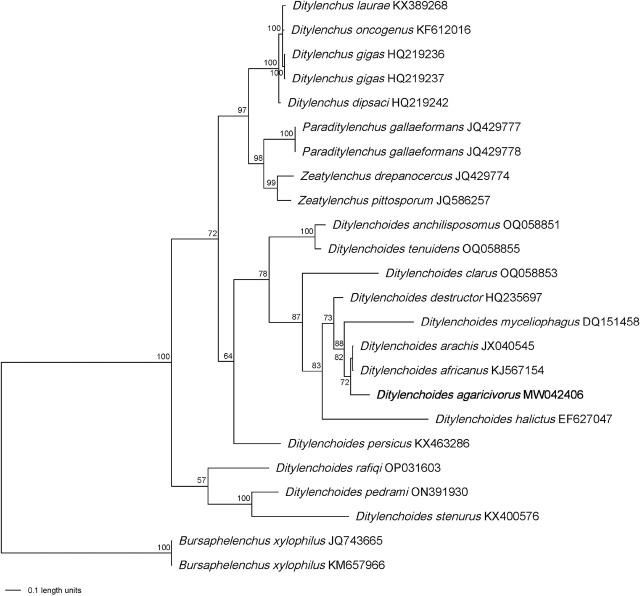
Bayesian tree generated from the ITS rRNA gene dataset of Anguinid nematodes with the GTR + I + G model. The newly obtained sequence is in bold letters (Scale bar = expected changes per site).

The Bayesian trees generated from D2D3 and ITS alignment by BI analysis are presented in Figures 3 and 4. After removing ambiguous sequences, the D2D3 expansion segment of 28S rRNA was 617 bp and then aligned with sequences of 27 ingroup and two outgroup taxa. Phylogenetic analysis revealed that *D. agaricivorus* n. sp. was grouped with *D. halictus* (AY589364) (Fig. 3). The sequence of ITS fragment after removing ambiguous sequences was 612 bp and was aligned with sequences of 21 ingroup and two outgroup taxa. *Ditylenchoides agaricivorus* n. sp. was grouped with *D. africanus* (KJ567154) and *D. arachis* (JX040545) (Fig. 4).

### Host test

The population of *D. agaricivorus* n. sp. increased after being cultured on the mycelium of plant pathogen *R. solani* and the economic importance of the mushroom *Agaricus bisporus*. The average Rf values of *R. solani* and *Agaricus bisporus* were 11.64 and 2.42, respectively. All nematodes recovered from *L. edodes*, *P. eryngii*, *V. volvacea* and *A. bitorquis* were adults and had an Rf value less than one, indicating no reproduction of *D. agaricivorus* n. sp. on these four mushrooms (Table 2).

**Table 2:** The average reproduction factor value of *Ditylenchoides agaricivorus* n. sp. recovered from fungal cultures, alfalfa seedlings, and callused carrot disks.

**Culture**	**Juvenile individuals**	**Adult individuals**	**Reproduction factor**
Callused carrot disk	0 ^a^	0	0
Alfalfa	0	0	0
*Rhizoctonia solani*	89.33 ± 31	143.56 ± 15	11.64 ± 2
*Lentinula edodes*	0	0.78 ± 1	0.04 ± 0
*Pleurotus eryngii*	0	0.56 ± 1	0.03 ± 0
*Volvariella volvacea*	0	1.89 ± 1	0.09 ± 0
*Agaricus bisporus*	13.22 ± 12	35.22 ± 11	2.42 ± 1
*Agaricus bitorquis*	0	1.89 ± 1	0.09 ± 0

a All numbers were in the form of mean ± standard deviation.

No nematodes were found in callused carrot disks and alfalfa cotyledon after staining the tissue with cotton blue, indicating that callused carrot disks and alfalfa failed to support the *D. agaricivorus* n. sp. population and that *D. agaricivorus* n. sp. was not able to invade into the plant tissue (Table 2).

## Discussion

As the first line of defense against pests and diseases, plant quarantine is important in preventing the international spread of pests and diseases. *Ditylenchoides agaricivorus* n. sp. was collected from coconut fiber used as growing media for staghorn ferns, intercepted during import quarantine, and had been identified because of quarantine requirements. A potential host test assessed the risk of *D. agaricivorus* n. sp. to agricultural production in Taiwan.

Many morphometrical characters of the genus *Ditylenchoides* have intraspecific variations, and only a few characters, such as the length of the stylet, the ratio of female body length to anal body diameter, the female tail length to anal body diameter ratio, and the spicule length are conserved enough at the species level to be used for identification (Hashemi et al., 2022). Recently, phylogenetic analyses have proven to be an effective method for separating genetically distinct species with common morphological characteristics (Subbotin et al., 2005; Vovlas et al., 2011).

*Ditylenchoides agaricivorus* n. sp. differs from other species in its morphological and morphometric characteristics and its phylogenetic status. Phylogenetic analyses of D2D3 expansion segments of 28S and ITS are both able to separate *D. agaricivorus* n. sp. from its sister species *D. africanus*, *D. arachis*, *D. destructor*, *D. halictus* and *D. myceliophagus*. Previous studies also support that the genus *Ditylenchoides* appears to be paraphyletic in these trees (Subbotin et al., 2006; Vovlas et al., 2011; Esmaeili et al., 2017). Although the *Ditylenchoides* sister species, including *D. agaricivorus* n. sp. shares several morphological features, such as rounded tail terminus, six lateral lines, and facultative fungal feeding habits like *D. arachis*, *D. destructor, D. halictus* and fungal feeding *D. myceliophagus* (Goodey, 1960; Giblin-Davis et al., 2010; Zhang et al., 2014), they are also different on some morphological traits such as female body length, stylet length, conus length, and post-vulval uterine sac length.

*Ditylenchoides* species were reported to be reared on several fungi, such as *Alternaria longipes* (Ellis and Everhart) Mason, *Botrytis cinerea* Persoon, *Monilinia fructicola* (Winter) Honey, and *Rhizoctonia cerealis* van der Hoeven (Wendt et al., 1995; Giblin-Davis et al., 2010; Zhang et al., 2014). *Ditylenchoides agaricivorus* n. sp. was grouped into the *Ditylenchoides* group phylogenetically and morphologically. Therefore, *D. agaricivorus* n. sp. probably shares their fungal feeding habits, and our results showed that *D. agaricivorus* n. sp. reproduces on *R. solani,* which provides another option for rearing mycophagus nematodes. *Rhizoctonia solani* is a fungal species that belongs to Basidiomycota (Dölfors et al., 2019), as well as several commercial mushroom *Lentinula edodes*, *Pleurotus eryngii*, *Volvariella volvacea* and *Agaricus bisporus* (Ukoima et al., 2009; Bederska-Łojewska et al., 2017). In the previous study, *D. destructor* and *D. myceliophagus* were observed to feed on mushroom hyphae (Goodey, 1960), and our potential host test showed that *D. agaricivorus* n. sp. could multiply on *A. bisporus*.

Alfalfa seedings and callused carrot disks were used for the mass production of *Ditylenchus dipsaci* (Kühn, 1857) Filipjev, 1936 and *Ditylenchus weischeri* Chizhov, Borisov and Subbotin, 2010, respectively (Krusberg, 1961; Hajihassani et al., 2017). Although multiplication of *D. destructor* on excised maize roots has been reported (Wendt et al., 1995), other *Ditylenchoides* species were reported to only feed on fungi (Wendt et al., 1995; Giblin-Davis et al., 2010; Zhang et al., 2014). Our results indicated that the feeding behavior and multiplication of *D. agaricivorus* n. sp. are different from other *Ditylenchus* species capable of feeding on plant tissue.

*Ditylenchoides destructor* and *Ditylenchus dipsaci* are commonly included in the quarantine lists of many countries (EPPO, 2024). In the past, we frequently encountered nematodes belonging to the genus *Ditylenchus and Ditylenchoides* in imported samples to Taiwan, with preliminary morphological identification indicating that most belong to the *Ditylenchoides*. To evaluate whether these nematodes have the potential to damage cultivated crops in Taiwan, we attempted to culture these *Ditylenchoides* nematodes from the quarantine samples using fungi. Among them, we successfully cultured a species of *Ditylenchoides* using *R. solani*, and we identified and evaluated the potential host test. In conclusion, we provided both morphological and molecular evidence to demonstrate that *D. agaricivorus* n. sp. is an undescribed species, which was recovered from growing media intercepted during the import of quaratine from Thailand. Phylogenic analyses revealed that *D. agaricivorus* n. sp was grouped with the *Ditylenchoides* species. Experimental tests also revealed that *D. agaricivorus* n. sp could feed on *A. bisporus*, which is an economically important mushroom. The quarantine risk of the *Ditylenchoides species* needs further studies to clarify.
